# Isotope Substitution of Promiscuous Alcohol Dehydrogenase Reveals the Origin of Substrate Preference in the Transition State

**DOI:** 10.1002/anie.201712826

**Published:** 2018-02-19

**Authors:** Enas M. Behiry, J. Javier Ruiz‐Pernia, Louis Luk, Iñaki Tuñón, Vicent Moliner, Rudolf K. Allemann

**Affiliations:** ^1^ School of Chemistry Cardiff University Park Place Cardiff CF10 3AT UK; ^2^ Departament de Química Física i Analítica Universitat Jaume I 12071 Castelló Spain; ^3^ Departament de Química Física Universitat de València 46100 Burjassot Spain

**Keywords:** alcohol dehydrogenase, enzymes, enzyme catalysis, enzyme models, isotope effects

## Abstract

The origin of substrate preference in promiscuous enzymes was investigated by enzyme isotope labelling of the alcohol dehydrogenase from *Geobacillus stearothermophilus* (BsADH). At physiological temperature, protein dynamic coupling to the reaction coordinate was insignificant. However, the extent of dynamic coupling was highly substrate‐dependent at lower temperatures. For benzyl alcohol, an enzyme isotope effect larger than unity was observed, whereas the enzyme isotope effect was close to unity for isopropanol. Frequency motion analysis on the transition states revealed that residues surrounding the active site undergo substantial displacement during catalysis for sterically bulky alcohols. BsADH prefers smaller substrates, which cause less protein friction along the reaction coordinate and reduced frequencies of dynamic recrossing. This hypothesis allows a prediction of the trend of enzyme isotope effects for a wide variety of substrates.

Significant insights into the role of enzyme motions in catalysis have recently been gained from investigations of isotopically labelled enzymes, in which the non‐exchangeable atoms ^12^C, ^14^N and ^1^H were replaced with ^13^C, ^15^N and ^2^H, respectively.^1^ Protein motions from femtosecond vibrations to millisecond structural changes are slowed by heavy‐isotope substitution, while the electrostatic properties remain unaffected, and thus the effect of protein motions on catalysis can be assessed.1e While this approach has revealed coupling of protein motions to substrate activation for a number of enzymes, a comparison of dihydrofolate reductases (DHFRs) from different extremophiles illustrated that dynamic coupling is minimized under physiological conditions.^2^ Indeed, for DHFRs, dynamic coupling is significant only when the reaction conditions are non‐physiological, where reorganizational motions are needed to facilitate efficient charge transfer.^2^, ^3^ Similarly, we postulated that the kinetic competence of promiscuous enzymes may be dependent on transition‐state stability in that dynamic coupling is reduced for fast substrates while it may be more significant for slower substrates.

To test this hypothesis, the enzyme kinetic isoptope effects for reactions catalyzed by the alcohol dehydrogenase from the *Geobacillus stearothermophilus* strain LLD‐R (BsADH) were measured (Figure 1). BsADH catalyzes the reversible oxidation of a wide variety of alcohols and has been shown to possess unique catalytic properties.^6^ Previously, the use of a bulky substrate benzyl alcohol in primary H/D substrate kinetic isotope effect (KIE) measurements revealed a “breakpoint” for the temperature dependence, where the KIE was largely temperature independent above 30 °C but increased sharply at lower temperatures.^7^ Site‐directed mutagenesis and hydrogen–deuterium exchange studies showed that the flexibility of a group of residues greatly affects the temperature dependence of the substrate KIE.6b, 7a,7b,7d It has been suggested that these residues are responsible for modifying the distance between hydride donor and acceptor.^8^ However, this has never been confirmed and it is unclear how a promiscuous enzyme such as BsADH can modify the donor–acceptor distances for a range of substrates.

**Figure 1:**
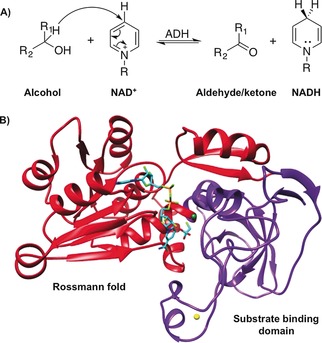
A) The reaction catalyzed by alcohol dehydrogenase (ADH). B) Cartoon representation of one subunit of BsADH (PDB ID: 1RJW),^4^ showing the substrate analogue trifluoroethanol and cofactor NAD^+^ in cyan, which are docked based on a homologue (PDB ID: 4GKV).^5^ The Rossmann fold (red), substrate‐binding domain (purple), and catalytic (green) and structural (yellow) Zn^2+^ ions are included.

The effect of dynamic coupling in BsADH catalysis for different substrates was investigated. Hydride transfer from benzyl alcohol to NAD^+^ during catalysis by BsADH is rate‐limiting under steady state conditions at pH 7.0. Hence, substrate KIEs (KIE=*k*_cat_^H^/*k*_cat_^D^) can be determined by measuring the steady‐state rate constant (*k*_cat_) using protiated and deuterated substrates. In agreement with previous work,6a, 7c the resulting substrate KIE is highly temperature‐dependent, increasing sharply at lower temperatures but remaining constant above 40 °C (Figure 2 A and Figures S1, S2 in the Supporting Information). The natural substrates of BsADH are generally small molecules, which serve as hydride acceptors under anaerobic conditions.^9^ Hence, the turnover rate constant for the oxidation of isopropanol was also measured and *k*_cat_ was noticeably higher than for benzyl alcohol (*k*_cat_=8.36±0.73 s^−1^ vs. 1.09±0.20 s^−1^ at 20 °C). The substrate KIE measured with protiated and deuterated isopropanol is temperature‐independent for the examined temperature range (20–50 °C), with an average value of 2.42±0.42 (Figure 2 A and Figures S1, S2).

**Figure 2:**
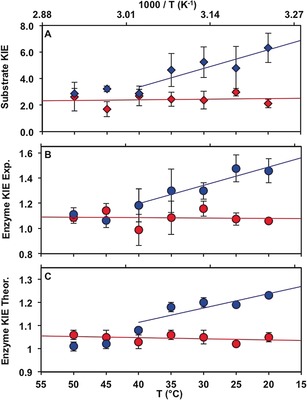
Temperature dependence of A) substrate kinetic isotope effect (KIE; *k*_cat_^H^/*k*_cat_^D^), B) experimental enzyme KIE (*k*_cat_^light BsADH^/*k*_cat_^heavy BsADH^), and C) QM/MM enzyme KIE (*γ* ^light BsADH^/*γ* ^heavy BsADH^). Results were obtained for the BsADH‐catalyzed oxidation of isopropanol (red) and benzyl alcohol (blue).

The kinetic difference between the two substrates was further explored by determining the enzyme kinetic isotope effects (*k*_cat_^light BsADH^/ *k*_cat_^heavy BsADH^). Expression of the BsADH cDNA in minimal medium containing ^13^C‐ and ^15^N‐labeled ingredients led to a 5.5 % molecular weight increase of the enzyme, thus suggesting that at least 99.8 % of the non‐exchangeable positions had been replaced by ^13^C and ^15^N (Figure S3; see the SI for CD and ESI‐MS analysis). For isopropanol, the rate constants for the “light” natural‐abundance (*k*_cat_^LE^) and “heavy” isotopically labeled (*k*_cat_^HE^) BsADH are statistically the same (*k*_cat_^LE^/*k*_cat_^HE^≈1) for all temperatures. Therefore, the activation parameters are indistinguishable for the reactions catalyzed by the “light” and the “heavy” enzymes. In contrast, when benzyl alcohol was the substrate, an enzyme KIE of 1.42±0.11 was determined at 20 °C. The enzyme KIE decreased gradually to unity as the temperature increased to 40 °C and stayed constant above this temperature. Due to the enzyme isotope sensitivity for the benzyl alcohol reaction, Δ*S* ^≠^ is significantly greater for the light enzyme when benzyl alcohol is used, and the activation free energy Δ*G* ^≠^ is noticeably lower at 20 °C (Table 1).

**Table 1:** Activation parameters during catalysis by light and heavy BsADH in 25 mm sodium phosphate buffer, pH 7.0: Δ*H* ^≠^, Δ*G* ^≠^ (in kcal mol^−1^), and Δ*S* ^≠^ (in kcal mol^−1^ K^−1^).

Substrate	Activation parameters	“Light” BsADH	“Heavy” BsADH
Isopropanol	Δ*H* ^≠a^	10.1±2.1	10.6±1.8
	Δ*S* ^≠a^	−19.1±0.5	−17.3±0.6
	Δ*G* ^≠^ at 20 °C	15.7±1.9	15.7±1.6
	Δ*G* ^≠^ at 40 °C	16.3±0.5	16.2±1.6
Benzyl alcohol	Δ*H* ^≠a^	12.7±0.8	15.3±0.5
	Δ*S* ^≠b^	−14.2±0.8	−6.1±1.8
	Δ*G* ^≠^ at 20 °C	16.9±0.6	17.1±0.1
	Δ*G* ^≠^ at 40 °C	17.2±0.6	17.2±0.1

[a] From 20–50 °C. [b] From 20–40 °C.

To explain the differences in the activation parameters, calculations for BsADH catalysis were carried out in ensemble‐averaged variational transition‐state theory (EA‐VTST) using QM/MM simulations. The rate constants of the chemical reaction were measured at different temperatures using Equation (1):^10^

(1) $$k\left(T\right)=\Gamma \left(T,\right)\frac{k_{B}T}{h}e^{-\left(\frac{{\Delta G}_{act}^{QC}\left(T,\xi \right)}{RT}\right)}=\frac{k_{B}T}{h}e^{-\left(\frac{{\Delta G}_{eff}\left(T\right)}{RT}\right)}$$

where ${\Delta G}_{act}^{QC}\left(T,\xi \right)$ is the quasiclassical activation free energy obtained from the classical mechanical (CM) potential of mean force (PMF) and including a correction for quantizing the vibrations orthogonal to the reaction coordinate (ξ ) and the vibrational free energy of the reactant mode that correlates with motion along the selected reaction coordinate (see the Supporting Information for details). *Γ*(*T*) is the temperature‐dependent transmission coefficient that can be expressed as:

(2) $$\Gamma \left(T,\xi \right)=\gamma \left(T,\xi \right)\kappa \left(T\right)$$

where $\gamma \left(T,\xi \right)$ is the recrossing transmission coefficient that corrects the rate constant for the trajectories that recross the dividing surface from the product valley back to the reactant valley, and *κ*(*T*) is the tunneling coefficient that accounts for reactive trajectories that do not reach the classical threshold energy. In Equation (1), ${\Delta G}_{eff}\left(T\right)$ is the effective activation free energy that incorporates both dynamic and quantum tunneling effects into the overall activation free energy. This can be compared to the activation free energies derived from the experimental rate constant (Table 1).

The rate constants for isopropanol and benzyl alcohol were computed at the seven temperature points. The ${\Delta G}_{eff}\left(T\right)$ values are in good agreement with the experimentally derived free‐energy barriers (${\Delta G}_{exp}$ ; Tables S4 and S5). The temperature dependences of the tunneling contributions (*κ*) are identical in the light and heavy enzyme for both benzyl alcohol and isopropanol (Tables S4, S5). This observation is in agreement with our previous computational and experimental studies, which indicated that tunneling or barrier modulation were not driven by compressive “promoting motions”.^2^, 3c, 11

The difference in the rate constants measured with the “light” and “heavy” enzyme are caused by changes in the recrossing coefficients γ .2b, 3b, 12 Indeed, because our simulations were carried out using an antisymmetric combination of the distances of the hydride to donor and the acceptor atoms as distinguished reaction coordinate, γ incorporates the effect of the remaining degrees of freedom of both the protein and substrate, which accounts for the additional friction observed on the distinguished reaction coordinate. Other protein motions apart from the ones involved in the reaction coordinate for successful reactive barrier crossing are reflected in the magnitude of the recrossing coefficient. Enzyme isotope substitution results in a reduction in protein motions, hence a larger friction on the advance of the system along the selected reaction coordinate. Accordingly, the theoretical values for the enzyme KIEs are similar to the experimental ones for both substrates (Figure 2 C and S8). This implies that an increase in the number of recrossing trajectories is observed and the value of the transmission coefficient is reduced with an enzyme KIE greater than 1 (γ ^LE^>γ ^HE^). The temperature dependence of the transmission coefficients is further analyzed in the Supporting Information.

Some of the normal modes of alcohols strongly couple to hydride transfer and depend on the substituents attached to the hydride donor (Figure 3 A). Since the step of hydride transfer is accompanied with a change in the hybridization state of the reacting carbon from *sp* ^3^ to *sp* ^2^, the substituents must align in the same plane as the reaction progresses, causing physical changes along the molecular skeleton of the entire substrate. Since the hydride transfer takes place within a narrow range of donor–acceptor distances, when benzyl alcohol is used, the bulky phenyl group substituent must be displaced. Indeed, the phenyl moiety of benzyl alcohol is motionally coupled to the movement of a number of active site residues (Val286, Thr40, Trp49, and Leu262) during the chemical transformation (Figure 3 A). While the average imaginary frequency for the hydride transfer in benzyl alcohol is about 540 cm^−1^, the bending of the phenyl substituent coupled to these residues has a smaller frequency (ca. 270 cm^−1^); this provokes additional recrossing events along the reaction coordinate due to the delay in the bending motion. Electrostatically, there is also significant charge delocalization due to the aromatic nature of the phenyl moiety. The degrees of freedom within the substrate are therefore directly coupled to the hydride transfer step and induce a larger friction. In contrast, these effects are less noticeable when isopropanol is used. The bending of the methyl groups occurs at a greater frequency (ca. 558 cm^−1^), which reduces the friction on the reaction coordinate. Also, the bending motion of the relatively small methyl groups in isopropanol hardly affects the surrounding residues (Figure 3 B). Hence, a recrossing coefficient closer to unity and weaker temperature dependence is obtained (Tables S4–S6).

**Figure 3:**
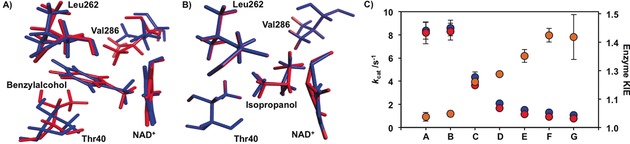
A, B) Normal mode associated to the substituents bending coupled to the hydride transfer in BsADH displayed as a superposition of two structures of the active site (red and blue) with benzyl alcohol (A) and isopropanol (B). C) The steady‐state rate constants (*k*_cat_) for “light” (blue) and “heavy” (red) BsADH, and enzyme kinetic isotope effect (orange) from substrate **A**–**G** at 20 °C and pH 7.0. The substrates are: isopropanol (**A**), 2‐butanol (**B**), ethanol (**C**), 1‐pentanol (**D**), cyclopentanol (**E**), and cinnamyl (**F**) and benzyl (**G**) alcohols.

The thermal activation of protein motions contributing to the observed enzyme KIE was also found to be substrate‐dependent (see the Supporting Information). As anticipated, the entropic barrier caused by the coupling of protein motions with hydride transfer ${(-T\Delta S}^{\neq }\left(\gamma \right))$ increases with temperature (see the Supporting Information for a detailed discussion). When a “bad” substrate with a low turnover rate constant such as benzyl alcohol is used, the barrier increases with temperature and this increase is more dramatic in the light enzyme than in the heavy counterpart (Table S6 and Figure S8). Eventually, the thermal energy of the protein reduces the difference in the activation of barrier crossing between the light and heavy enzymes, which leads to an enzyme KIE closer to unity at higher temperature. Although BsADH is known to be promiscuous, the active site is not designed to accommodate “uncommon” substrates that provoke substantial displacement. Indeed, most bacterial ADHs are used to transfer hydride to small carbonyl substrates under anaerobic conditions.^9^ Previously, the temperature breakpoint for the substrate kinetic isotope effect was shown to disappear when Trp87 in the substrate binding pocket was replaced with alanine.7d

To confirm for theory of “good” and “bad” substrates, two groups of substrates were used; one contains small, non‐conjugated alcohols including isopropanol, 2‐butanol, ethanol and 1‐pentanol, and the other contains bulky and/or highly conjugated systems such as cyclopentanol, cinnamyl and benzyl alcohols (Figure 3 C). Small, non‐conjugated substrates are “good” substrates, resulting in higher *k*_cat_ constants than those for the bulky ones. In line with our proposal,3b, 13 dynamic coupling was found to be less significant for small, non‐conjugated substrates at 20 °C (*k*_cat_^LE^/*k*_cat_^HE^≤1.2; Figure 3 C). Sampling an ideal configuration for hydride transfer is likely less energetically demanding for “good” substrates where less reorganization of the active site residues is required, and thus they lead to higher values of *k*_cat_ and lower enzyme KIEs (Figure 3 C and Tables S8 and S9). Interestingly, at physiological temperature, the enzyme KIE remains close to unity irrespective of the substrate used (Table S9). This may represent adaptation of the enzymes to their natural environment, indicating that, once a preorganized active site is generated, protein motions are not essential for the chemical step.

In summary, for promiscuous enzymes such as BsADH, “good” substrates induce fewer recrossing events along the antisymmetric reaction coordinate due to efficient electrostatic preorganization. In contrast, “bad” substrates cause substantial active‐site reorganization coupled to the substituents of the substrate, and electrostatic preorganization is suboptimal. Enzymes may have evolved to reduce unwanted friction in the transition states, which may help in the protein engineering of ADHs to generate useful products.^14^

## Conflict of interest

The authors declare no conflict of interest.

## Supporting information

As a service to our authors and readers, this journal provides supporting information supplied by the authors. Such materials are peer reviewed and may be re‐organized for online delivery, but are not copy‐edited or typeset. Technical support issues arising from supporting information (other than missing files) should be addressed to the authors.

Supplementary
