# Supplementary material for: Isotope Substitution of Promiscuous Alcohol Dehydrogenase Reveals the Origin of Substrate Preference in the Transition State
Source: Angew Chem Int Ed Engl. 2018 Feb 19;57(12):3128–31. doi: 10.1002/anie.201712826 (PMC5861672; doi:10.1002/anie.201712826)
Supplement: Supplementary file 1 — Supplementary [file ANIE-57-3128-s001.pdf]

Angewandte Chemie
Eine Zeitschrift der Gesellschaft Deutscher Chemiker

Supporting Information

Isotope Substitution of Promiscuous Alcohol Dehydrogenase Reveals the Origin of Substrate Preference in the Transition State

Enas M. Behiry, J. Javier Ruiz-Pernia, Louis Luk, Iñaki Tuñón, Vicent Moliner,* and Rudolf K. Allemann**

anie_201712826_sm_miscellaneous_information.pdf

Experimental work

Chemicals. NAD^+ was bought from Sigma, BsADH plasmid from, isopropanol and d_6-isopropanol from Fisher Scientific. All other alcohol substares were purchased from Sigma-Aldrich or Lancaster. α,α-d_2-benzyl alcohol was either purchased from Sigma-Aldrich or synthesized by reducing benzoic acid using $NaBD_4$.[1] All the substrates were distilled before being used for kinetics measurements.

Enzyme expression and purification. The BsADH plasmid was transformed into BL21(RP) competent cells and expressed at 16 °C, either in LB media (for light enzyme) or minimal media containing ^{13}C glucose and ^{15}N ammonium chloride (for the heavy enzyme). The enzyme was purified in 50 mM sodium phosphate buffer containing 5 mM 2-mercabtoethanol, pH 7, using Q-sepharose anion exchange column then loaded into cibacron blue affinity column. The enzyme was eluted from the final step using NAD^+ then stored at 4 °C. A Superdex G25 column was used to remove the cofactor NAD^+ before running kinetics. Purity and mass of the enzyme were confirmed by SDS-PAGE and LC-MS.

Staedy state Kinetics. Kinetics has been performed on Jasco 2660 UV spectrometer in 25 mM sodium phosphate buffer, pH 7. The cofactor NAD^+ concentration has been determined at 260 using extension coefficient 17800 $M^{-1}cm^{-1}$. Enzyme concentration has been determined at 210, 215 and 220 nm using the extension coefficient 20, 15 and 11 mg/ml. Kinetics have been measured in the temperature range from 20 to 50 °C due to low solubility of benzyl alcohol in water below 20 °C. For each data point at least four substrate concentrations and cofactor concentrations has been used, then the data was fitted to the two substrate equation:

$$V = \frac{k_{cat}\,[E]\,[A]\,[B]}{K_{ia}\,K_m^B + K_m^A\,[B] + K_m^B\,[A] + [A]\,[B]}$$

Then, V and k_{cat} were calculated using non-linear fitting, where V is the velocity k_{cat} is the steady state turn over number, $[E]$ is the enzyme concentration, $[A]$ and $[B]$ are the cofactor NAD^+ and the substrate concentration, respectively, K_{ia} is the dissociation constant of the E.NAD^+ binary complex and K_m^A and K_m^B are the Michaelis constants for the cofactor NAD^+ and the substrate respectively.

For the enzyme kinetic isotope effect, the kinetics of the 'light' and 'heavy' enzymes have been performed at the same time and all the experiments has been repeated at least three times, then errors have been calculated form the data standered deviations.

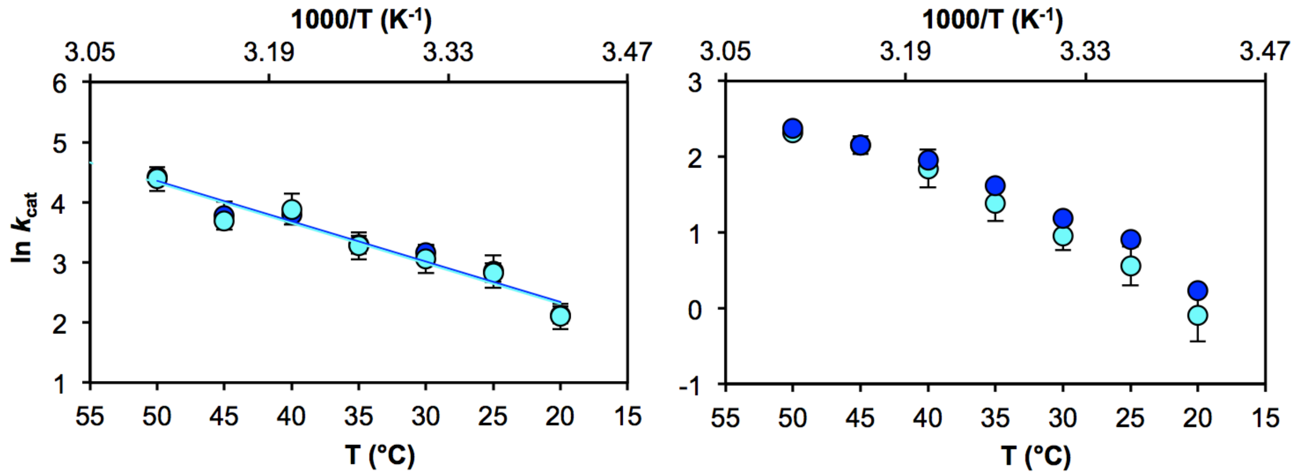

Figure S1: Arrhenius plots for "light" BsADH (blue) and "heavy" ^{13}C, ^{15}N-BsADH (cyan) with isopropanol (left) and benzyl alcohol (right).

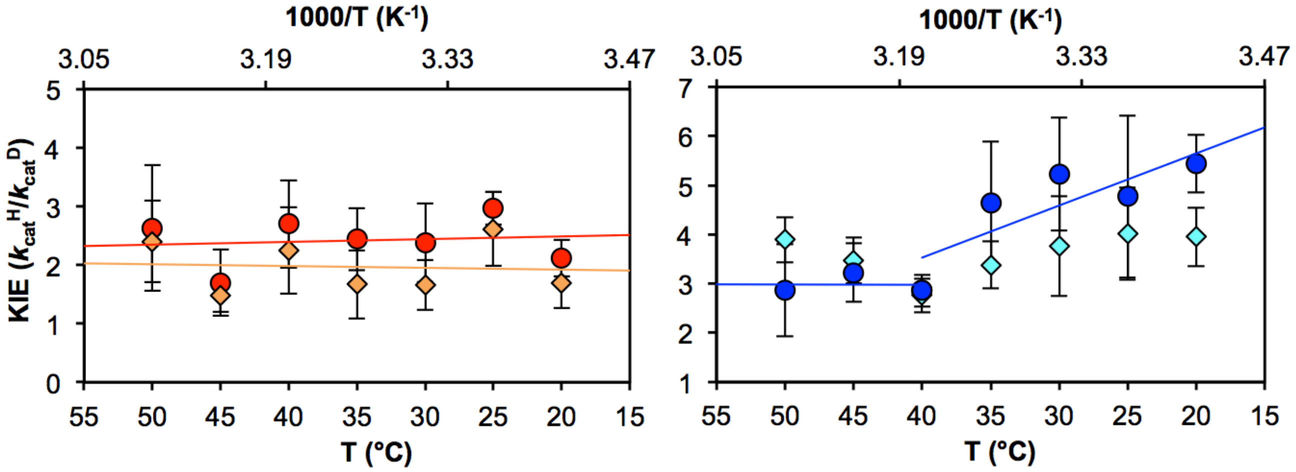

Figure S2: Temperature dependences of substrate kinetic isotope effects for "light" BsADH (circles) and "heavy" 13C, 15N-BsADH (diamonds) with isopropanol (left) and benzyl alcohol (right)

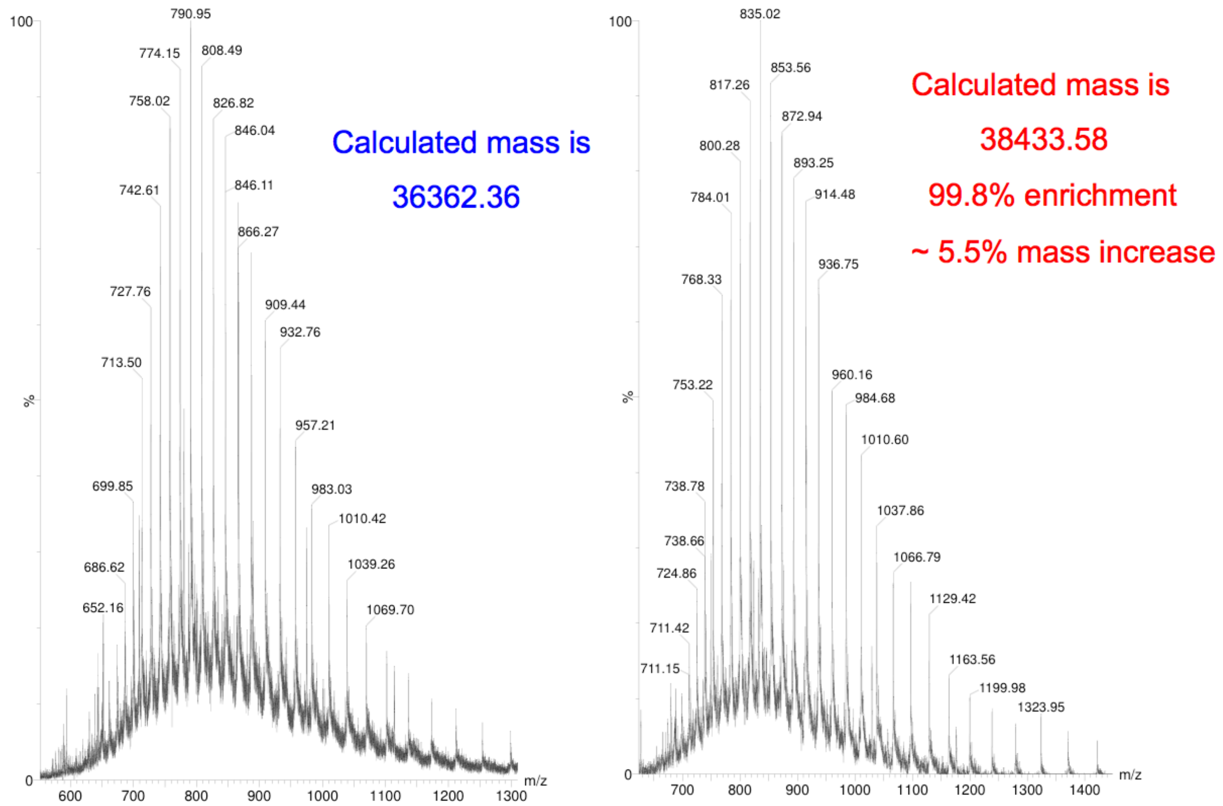

Figure S3: LC-MS of light and ^{13}C, ^{15}N-Heavy BsADH showing mass increase of ~5.5 % in the heavy enzyme and ensuring 99.8 % labelling of all the non-exchangeable positions.

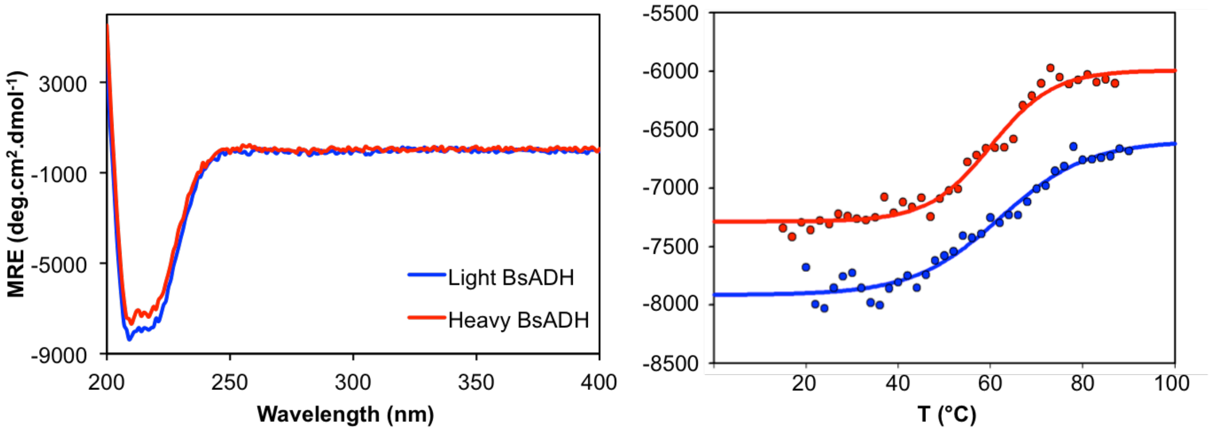

Figure S4: CD spectrum at 20 °C, (Left), and effect of temperature on the circular dichroism signals at 217 nm (Right) for Light BsADH (blue) and ^{13}C, ^{15}N-BsADH (red) in 10 mM sodium phosphate buffer. The melting points for both light and heavy enzymes are determined to be 60.1 ± 1.0 and 62.1 ± 1.6 respectively, using sigma plot.

Table S1: Steady state rate constants and substrate kinetic isotope (SKIE) effect during catalysis by light and heavy BsADH with isopropanol

T (°C)	^{12}C, ^{14}N-BsADH			^{13}C, ^{15}N-BsADH		
	k_{cat}^{H}	k_{cat}^{D}	*SKIE*	k_{cat}^{H}	k_{cat}^{D}	*SKIE*
	(s^{-1})		(k_{cat}^{H}/ k_{cat}^{D})	(s^{-1})		(k_{cat}^{H}/ k_{cat}^{D})
20	8.36 ± 0.73	3.95 ± 0.41	2.12 ± 0.17	8.18 ± 0.95	5.30 ± 0.66	1.69 ± 0.43
25	17.29 ± 2.46	5.83 ± 0.44	2.97 ± 0.28	16.92 ± 2.65	6.35 ± 0.17	2.61 ± 0.63
30	23.62 ± 6.43	9.93 ± 4.02	2.38 ± 0.67	21.20 ± 4.94	13.20 ± 1.56	1.66 ± 0.43
35	26.83 ± 3.10	11.00 ±1.73	2.44 ± 0.53	26.35 ± 5.83	15.76 ± 5.44	1.67 ± 0.58
40	44.60 ± 6.68	16.52 ± 4.65	2.70 ± 0.75	48.64 ± 12.71	21.66 ± 0.63	2.25 ± 0.74
45	43.67 ± 3.97	25.69 ± 12.70	1.70 ± 0.57	40.01 ± 3.43	27.00 ± 4.15	1.48 ± 0.28
50	83.46 ± 19.34	31.72 ± 16.33	2.63 ± 1.07	80.34 ± 15.99	33.43 ± 7.85	2.40 ± 0.70

Table S2: Steady state rate constants and substrate kinetic isotope effect during catalysis by light and heavy BsADH with benzyl alcohol

T (°C)	^{12}C, ^{14}N-BsADH			^{13}C, ^{15}N-BsADH		
	k_{cat}^{H}	k_{cat}^{D}	*SKIE*	k_{cat}^{H}	k_{cat}^{D}	*SKIE*
	(s^{-1})		(k_{cat}^{H}/ k_{cat}^{D})	(s^{-1})		(k_{cat}^{H}/ k_{cat}^{D})
20	1.09 ± 0.20	0.20 ± 0.05	5.44 ± 0.59	0.77 ± 0.18	0.19 ± 0.02	3.95 ± 0.60
25	2.47 ± 0.61	0.52 ± 0.28	4.77 ± 1.65	1.74 ± 0.45	0.43 ± 0.13	4.01 ± 0.94
30	3.26 ± 0.75	0.62 ± 0.19	5.23 ± 1.15	2.60 ± 0.50	0.74 ± 0.32	3.76 ± 1.01
35	5.02 ± 1.55	1.08 ± 0.37	4.64 ± 1.24	3.99 ± 0.94	1.35 ± 0.46	3.38 ± 0.48
40	7.07 ± 1.00	2.48 ± 0.32	2.86 ± 0.32	6.32 ± 1.56	2.29 ± 0.11	2.76 ± 0.35
45	8.65 ± 0.53	2.69 ± 0.25	3.22 ± 0.21	8.59 ± 0.99	2.46 ± 0.10	3.47 ± 0.47
50	10.69 ± 0.38	2.81 ± 0.03	3.59 ± 0.04	10.08 ± 0.16	2.59 ± 00.53	3.89 ± 0.46

**Table S3: Temperature dependence of the enzyme kinetic isotope effect (EKIE) for both isopropanol and benzyl alcohol during catalysis by BsADH in 25 mM sodium phosphate buffer, pH 7.**		
**T (°C)**	**EKIE** ($k_{cat}^{light\ BsADH}$ / $k_{cat}^{heavy\ BsADH}$)	
	**Isopropanol**	**Benzyl alcohol**
20	1.02 ± 0.02	1.42 ± 0.11
25	1.02 ± 0.05	1.43 ± 0.11
30	1.11 ± 0.06	1.25 ± 0.07
35	1.04 ± 0.13	1.25 ± 0.17
40	0.94 ± 0.12	1.13 ± 0.13
45	1.09 ± 0.06	1.01 ± 0.05
50	1.04 ± 0.05	1.06 ± 0.05

Computational Details

The simulation model. The starting structure for dynamic simulations of ADH was obtained from the Protein Data Bank entry 1RJW[2] which codes for the crystal structure of the enzyme bounded to the inhibitor trifluoroethanol. The substrate isopropanol was introduced instead of the inhibitor while the cofactor NAD^+ was introduced manually based on the coordinates of the ligand-bound of other ADH with and entry 1ADC.[3] The PROPKA3 program[4] was employed to estimate the *pK*a values of the titratable protein residues to verify their protonation states at pH 7. To neutralize the system, 2 sodium counterions were placed in optimal electrostatic positions around the enzyme. Finally, the system was solvated using a cubic box of TIP3P water molecules with side lengths of 130.0 Å; water molecules with an oxygen atom within 2.8 Å of any heavy atom were removed.

The full systems were formed by 176206 atoms when isopropanol was the substrate, 176210 for benzyl alcohol and 176203 for ethanol. The model contained the protein (1356 residues for the whole tetramer; thus, 339 residues per subunit plus 8 zinc atoms) with 20664 atoms, the substrate and cofactor (70 atoms), 2 sodium ions and 51817 water molecules, 332 observed in the crystal structure and 51485 added during solvation (a total of 155451 atoms). After 500 minimization steps using a conjugate gradient method, 10 ns of molecular dynamics simulations using NAMD[5] software were carried out in order to equilibrate the system at 298 K. In the following simulations the whole system was divided into a QM part and a MM part to perform combined QM/MM calculations (Figure 1). The quantum subsystem contained 61 atoms, including the full substrate, the zinc ion and parts of the cofactor (nicotinamide ring and the ribose), Cys38, Cys148 and His61. Four hydrogen 'link' atoms[6] were used to saturate the valence at the QM-MM boundary (Figure 1). The quantum atoms were treated by the AM1 Hamiltonian,[7] modified using specific reaction parameters (denoted as AM1-SRP) developed previously for DHFR.[8] The protein atoms and the ions were described by OPLS-AA[9] force field while the water molecules were described by the TIP3P potential.[10] Cutoffs for the nonbonding interactions were applied using a switching function within a radius range of 13.0 to 9.0 Å. All the molecules further than 25 Å from the substrate were frozen, while the rest of the system was allowed to move. From the final structure, the substrate was substituted for benzyl alcohol and ethanol. After, 5000 steps of QM/MM minimization followed by 200 ps of QM/MM simulation at each temperature: 293, 298, 303, 308, 313, 318 and 323 K, for isopropanol and benzyl alcohol and only one at 293 K for ethanol. Heavy ADH was prepared *in silico* by modifying the masses of all ^{14}N, ^{12}C and non-exchangeable ^{1}H atoms to those of ^{15}N, ^{13}C and ^{2}H, respectively. The ratio between the molecular weights of the simulated heavy and light enzymes was 1.114, similar to the experimentally measured molecular weight increase.

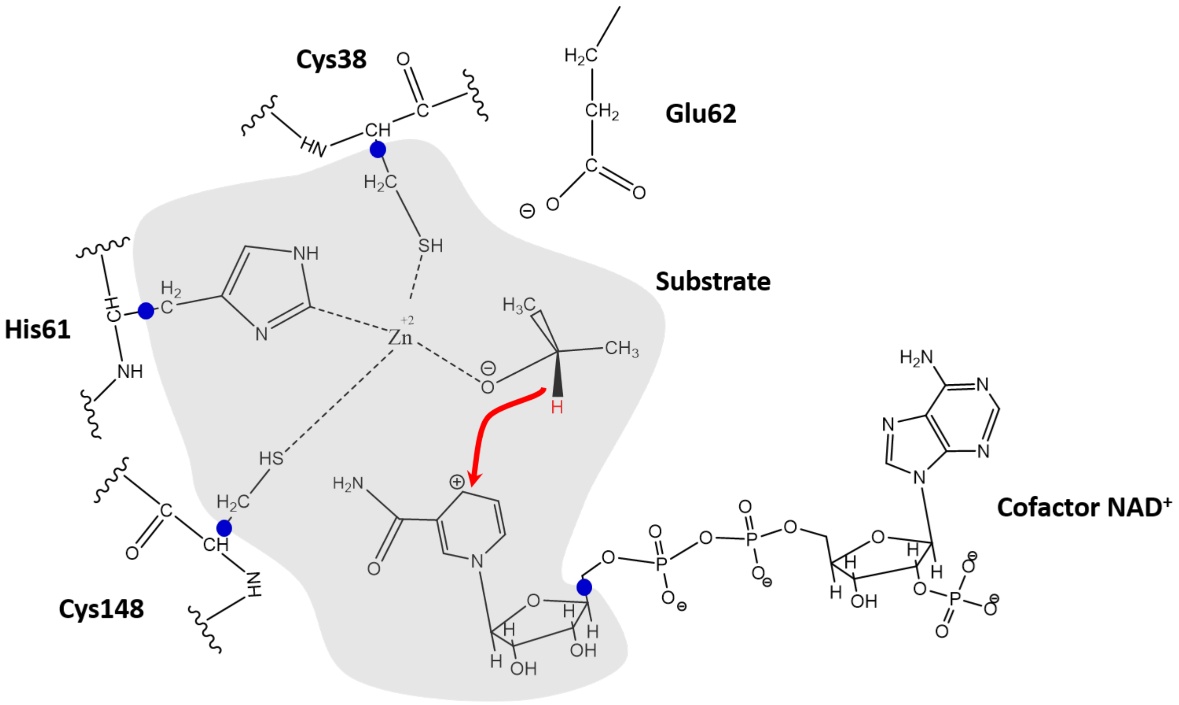

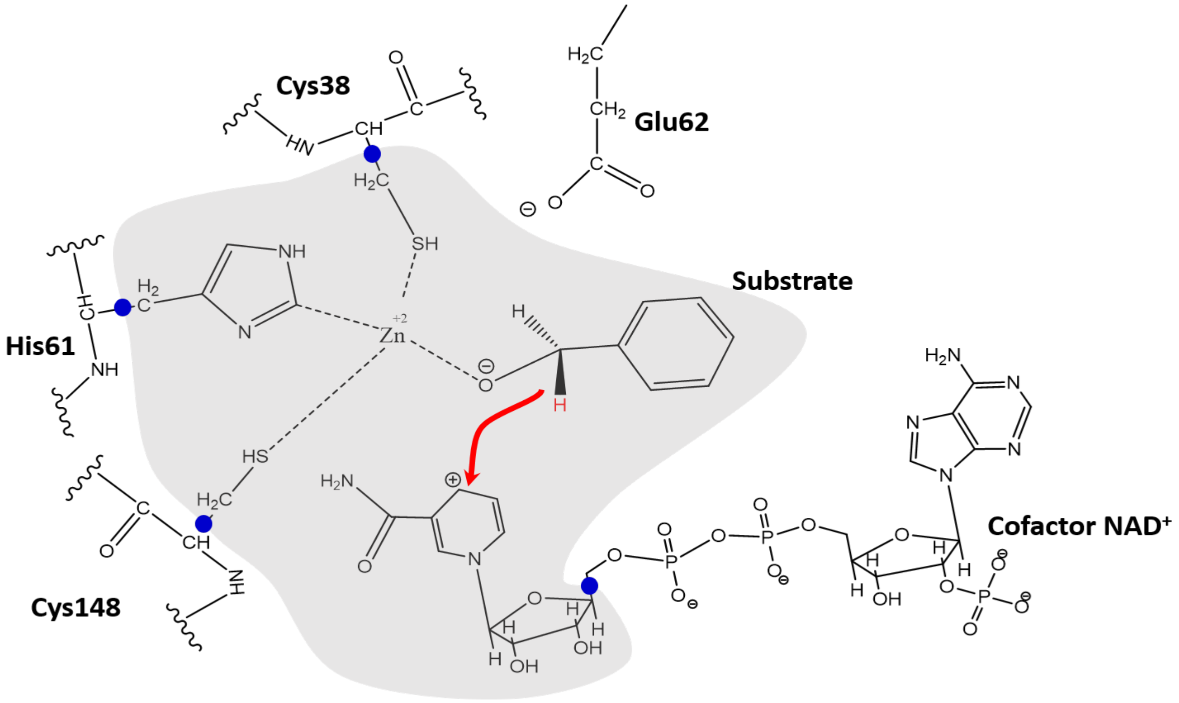

Figure S5. Schematic representation of the active site and definition of the QM/MM subsystem for the substrate isopropanol (top) and benzyl alcohol (bottom). QM-MM frontier link atoms are indicated as blue dots.

Potentials of Mean Force (PMF).[11] From the final structures of the QM/MM simulations at each temperature, one-dimensional PMFs, W^{CM}, were computed using the antisymmetric combination of distances describing the hydride transfer, $\xi = d_{C1Ht}-d_{HtC4}$, as the reaction coordinate. The umbrella sampling approach was used,[12] with the system restrained to remain close to the desired value of the reaction coordinate by means of the addition of a harmonic potential with a force constant of 2500 kJ mol^{-1} A^{-2} , which allows good overlap between windows. The reaction coordinate was then explored in a range from –1.93 to 1.36 Å and the total number of windows was 48. The probability distributions obtained from MD simulations within each individual window were combined by means of the weighted histogram analysis method (WHAM).[13] 100 ps of relaxation and 100 ps of production MD, with a time step of 0.5 fs, in the canonical ensemble (NVT, with reference temperatures at 293, 298, 303, 308, 313, 318 and 323 K) and the Langevin–Verlet integrator,[14] were used in the simulations.

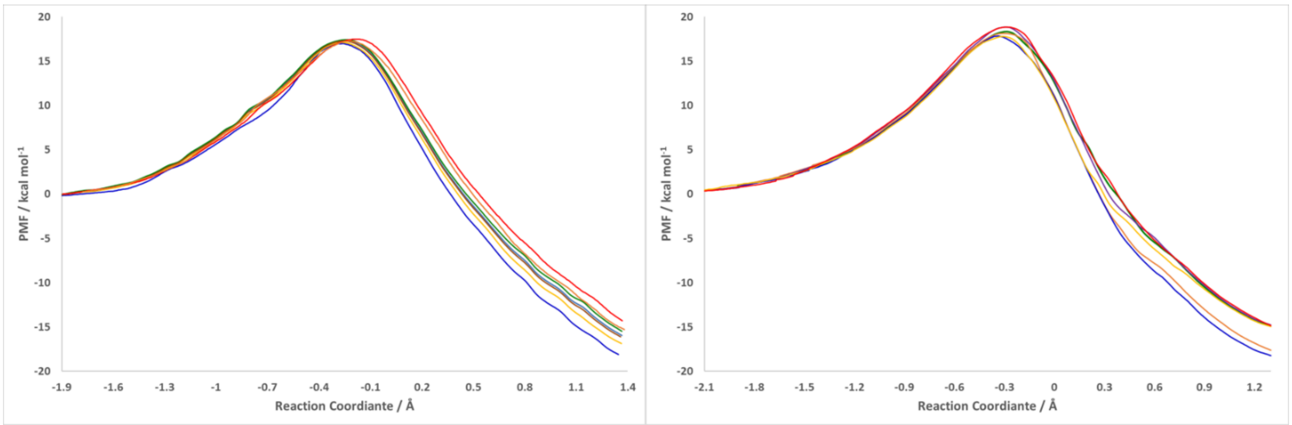

Figure S6. Classical mechanical AM1-SRP/MM potential of mean force (PMF) obtained for hydride transfer catalyzed by BsADH with isopropanol (left panel) and benzyl alcohol (right panel) at 293 K (blue line), 298 K (yellow line), 303 K (brown line), 308 K (green line), 313 K (orange line), 318 K (mauve line) and 323 K (red line). The reaction coordinate corresponds to the antisymmetric combination of distances between the hydride and the donor and acceptor carbon atoms.

Calculation of the recrossing transmission coefficient. Grote-Hynes (GH) theory can be applied to describe the evolution of the system along the reaction coordinate at the TS. In particular, the transmission coefficient can be obtained as the ratio between the reactive frequency and the equilibrium barrier frequency:[15]

$$\gamma_{GH} = \frac{\omega_r}{\omega_{eq}} \quad \text{(S1)}$$

with the equilibrium frequency derived from a parabolic fit of the PMF around the free energy maximum and the reactive frequency ω_r is obtained via the GH equation:[16]

$$\omega_r^2 - \omega_{eq}^2 + \omega_r \int_0^{\infty} \zeta_{TS}(t) e^{-\omega_r t} dt = 0 \quad \text{(S2)}$$

$\zeta_{TS}(t)$ is the friction kernel obtained at the TS, assuming that recrossings take place in the proximity of this dynamic bottleneck:[16b, 17]

$$\zeta(t) = \frac{\langle F_{RC}(0) F_{RC}(t) \rangle}{\mu_{RC} k_B T} \quad \text{(S3)}$$

where $F_{RC}(t)$ is the force on the reaction coordinate and μ_{RC} the associated reduced mass. For the evaluation of the TS friction kernel at different temperatures, the system in the reactant state was further equilibrated by means of 200 ps of classical MD at 293, 298, 303, 308, 313, 318 and 323 K. Then, by means of QM/MM MD simulations, we determined the one dimensional PMF as a function of the reaction coordinate in the vicinity of the transition state region at each temperature using the fDYNAMO library.[18] 50 ps (10 ps of relaxation and 40 ps of production) of restrained QM/MM MD simulations were run at windows of the PMFs. Finally, a QM/MM MD simulation of 20 ps was performed at the top of the PMF constraining the reaction coordinate at the corresponding value. With this purpose a time-step of 0.1 fs was used to ensure the convergence of the algorithm. Forces acting on the reaction coordinate were saved at each simulation step. We previously tested that the GH approach gives transmission coefficients in very good agreement with those obtained from activated trajectories initiated at the TS ensemble.[19],[20] The recrossing transmission coefficients $\gamma(T,\xi)$ were calculated using eq. S1 for the light and heavy versions of ADH. Because the heavy enzyme has larger mass some of its internal motions are slower. Then, this version of the enzyme presents a higher friction and thus a smaller value of the transmission coefficient. The transmission coefficients of the two versions were found to be statistically different.

Temperature dependence of the recrossing transmission coefficient The recrossing transmission coefficient can be further analyzed in terms of an entropic contribution to the barrier:

$$\Delta S^{\ddagger}(\gamma) = R \cdot \ln(\gamma) + \frac{RT}{\gamma} \cdot \frac{\partial \gamma}{\partial T} \quad \text{(S4)}$$

As $\gamma < 1$, the first term $R \cdot \ln(\gamma)$ in Eq. S4 is negative because of the contribution of substrate and protein motions, leading to an increase of free energy in barrier crossing. These degrees of freedom must reach a particular value at the transition state, such that the entropy of the system is reduced with respect to the equilibrium description. According to our computed values (see SI), this barrier is always larger in the isotopically labeled version of BsADH for both substrates. The second term $\frac{RT}{\gamma} \cdot \frac{\partial \gamma}{\partial T}$ of Eq. S4 incorporates additional friction due to the thermal activation of

motions coupled to the hydride antisymmetric stretch. In the case of the BsADH reactions, this term $\frac{RT}{\gamma} \cdot \frac{\partial\gamma}{\partial T}$ is negative and thus thermal activation contributes to an increase of the mass-dependent entropic barrier (SI, Table S6).

For isopropanol, the transmission coefficient in the heavy enzyme is slightly lower than that in the light counterpart at all the temperatures analyzed (SI Figure S8). This is likely due to the decrease in the frequencies of the protein motions and an increase in the friction on the reaction coordinate. The transmission coefficients for both light and heavy enzymes decrease smoothly with temperature; the resulting slopes $\frac{\partial\gamma}{\partial T}$ are therefore similar in magnitude (SI, Figure S8 and Table S6). The measured enzyme KIE is only slightly larger than unity and also largely temperature independent (Figure 2C). In contrast, when benzyl alcohol is used, a significantly different observation was made. The recrossing coefficients γ in both the light and heavy enzymes decrease with temperature, but the light enzyme shows a steeper decrease (i.e. a greater magnitude of $\frac{\partial\gamma}{\partial T}$, SI, Figure S8 and Table S6). Particularly, at low temperatures the recrossing coefficients computed in the light enzyme are significantly larger than those of the heavy enzyme (SI, Figure S8). Accordingly, the computational enzyme KIE (Figure 2C) is temperature dependent and large at low temperatures.

Ensemble Averaged Variational Transition State Theory. Acording to the Ensemble-Averaged Variational Transition State Theory (EA-VTST),[21] the theoretical estimation of the rate constant can be obtained as shown in Eq 1. of the manuscript. ΔG_{act}^{QC} is the quasiclassical activation free energy at the transition state, obtained from the classical mechanical (CM) PMF ($W^{CM}(T, \xi)$) as:

$$\Delta G_{act}^{QC} = [W^{CM}(T,\xi^*) + \Delta W_{vib}(T,\xi^*)] - [W^{CM}(T,\xi_R) + \Delta W_{vib,R}(T) + G_{R,T,F}^{CM}] \quad \text{(S4)}$$

where $\Delta W_{vib}(T, \xi^*)$ corrects $W^{CM}(T, \xi^*)$ to account for quantized vibrations orthogonal to the reaction coordinate along which the PMF is defined, ξ at the maximum of the PMF, ξ*; $\Delta W_{vib,R}(T)$ corrects $W^{CM}(T, \xi_R)$ for quantized vibrations at the reactant side minimum of the PMF, ξ_R, and $G^{CM}_{R,T,F}$ is a correction for the vibrational free energy of the reactant mode that correlates with motion along the reaction coordinate.[21a]

To correct the classical mechanical PMF, W^{CM}, normal mode analyses were performed for the quantum region atoms. To perform these calculations we localized 15 TS structures starting from different configurations of the corresponding simulation windows. After tracing the minimum energy path, we optimized 15 reactant structures and obtained the Hessian matrix for all the stationary structures.[22] The final quantum mechanical vibrations correction to the quasi-classical activation free energy was obtained as an average over these structures.

The tunneling transmission coefficients were calculated with the small-curvature tunneling (SCT) approximation, which includes reaction-path curvature appropriate for enzymatic hydride transfers. The final tunneling contribution (see Tables S4 and S5) is obtained as the average over the reaction paths of 15 TS structures, as made in our previous works.[20, 23]

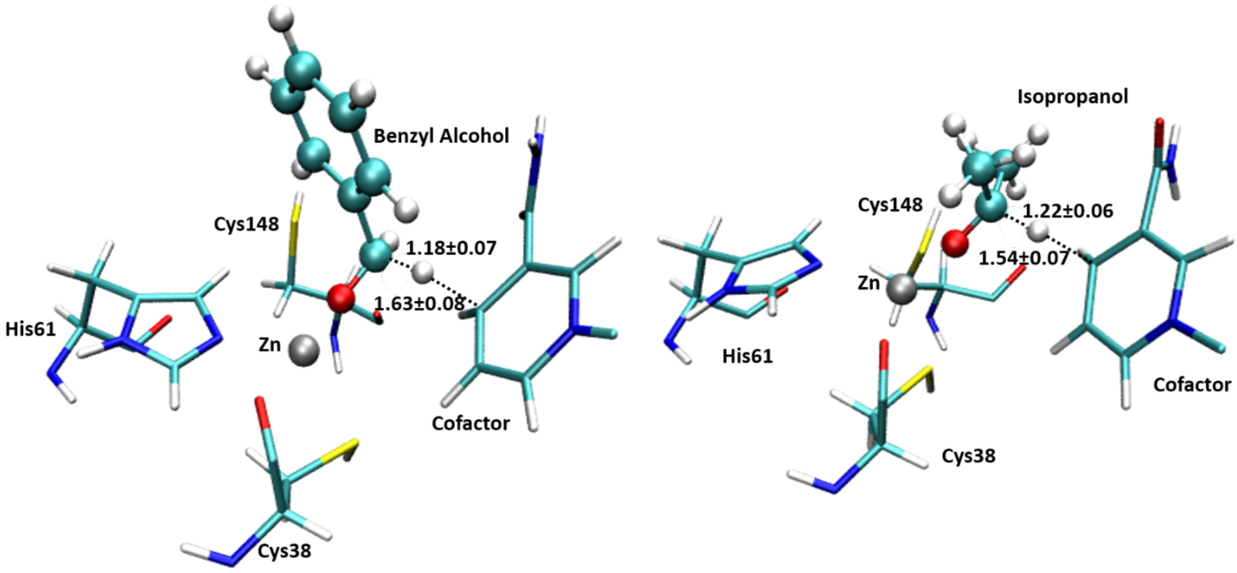

Figure S7. Transition structures for the hydride transfer in BsADH from benzyl alcohol (left) and isopropanol (right). The values of the donor-hydride and acceptor-hydride distances are averaged from the 15 localized transition state structures.

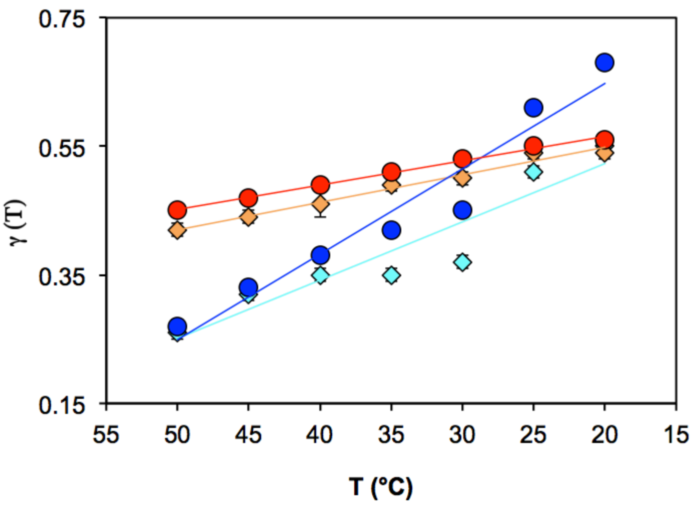

Figure S8. QM/MM results of the temperature dependence of transmission coefficients, obtained for isopropanol (red and orange) and benzyl alcohol (blue and cyan) in the light (circles) and heavy (diamonds) versions of BsADH.

Table S4. Temperature dependence of the recrossing transmision coeficien, γ, the tunneling coefficient, κ, the quasiclassical activation free energy, ΔG_{ACT}^{QC}, and the effective phenomenological activation free energy, ΔG_{eff}, and Enzyme Kinetic Isotope Effects, $EKIE_{theo}$, determined by QM/MM calculations using isopropanol as substrate. The free energy barriers deduced from the experimental rate constant within the TST framework, ΔG_{exp} , and $EKIE_{exp}$ are listed for comparative purposes.

T (K)	Enzyme	γ	κ	ΔG_{ACT}^{QC} (kcal mol^{-1})	ΔG_{eff} (kcal mol^{-1})	ΔG_{exp} (kcal mol^{-1})	$EKIE_{theo}$	$EKIE_{exp}$
293	Light	0.56 ± 0.01	4.4 ± 0.3	15.38 ± 0.92	14.85 ± 1.07	15.72	1.05 ± 0.02	1.01 ± 0.01
	Heavy	0.54 ± 0.01	4.3 ± 0.3	15.37 ± 0.96	14.88 ± 1.12	15.72		
298	Light	0.55 ± 0.01	3.8 ± 0.5	15.29 ± 1.02	14.86 ± 1.27	15.76	1.02 ± 0.01	1.02 ± 0.05
	Heavy	0.54 ± 0.01	3.7 ± 0.4	15.28 ± 1.06	14.87 ± 1.26	15.78		
303	Light	0.53 ± 0.01	3.4 ± 0.6	15.60 ± 0.82	15.24 ± 1.12	15.85	1.05±0.03	1.11 ± 0.06
	Heavy	0.50 ± 0.01	3.4 ± 0.5	15.59 ± 0.86	15.28 ± 1.11	15.92		
308	Light	0.51 ± 0.01	3.0 ± 0.7	15.21 ± 0.72	14.95 ± 1.07	16.04	1.06 ± 0.02	1.04 ± 0.13
	Heavy	0.49 ± 0.01	2.9 ± 0.6	15.20 ± 0.76	14.98 ± 1.06	16.06		
313	Light	0.49 ± 0.01	2.8 ± 0.7	14.92 ± 0.72	14.72 ± 1.07	16.29	1.03 ± 0.03	0.94 ± 0.12
	Heavy	0.46 ± 0.02	2.8 ± 0.5	14.90 ± 0.76	14.74 ± 1.01	16.24		
318	Light	0.47± 0.01	2.6 ± 0.6	14.93 ± 0.82	14.81±1.12	16.28	1.05 ± 0.03	1.09 ± 0.06
	Heavy	0.44 ± 0.01	2.6 ± 0.5	14.92 ± 0.86	14.84 ± 1.11	16.33		
323	Light	0.45 ± 0.01	2.5 ± 0.7	14.75 ± 0.82	14.68 ± 1.17	16.13	1.06 ± 0.02	1.04 ± 0.05
	Heavy	0.42 ± 0.01	2.4 ± 0.6	14.73 ± 0.86	14.72 ± 1.16	16.15		

Table S5. Temperature dependence of the recrossing transmision coeficien, γ, the tunneling coefficient, κ, the quasiclassical activation free energy, ΔG_{ACT}^{QC}, and the effective phenomenological activation free energy, ΔG_{eff}, and Enzyme Kinetic Isotope Effects, $EKIE_{theo}$, determined by QM/MM calculations using benzyl alcohol as substrate. The free energy barriers deduced from the experimental rate constant within the TST framework, ΔG_{exp} , and $EKIE_{exp}$ are listed for comparative purposes.

T (K)	Enzyme	γ	κ	ΔG_{ACT}^{QC} (kcal mol^{-1})	ΔG_{eff} (kcal mol^{-1})	ΔG_{exp} (kcal mol^{-1})	$EKIE_{theo}$	$EKIE_{exp}$
293	Light	0.68 ± 0.01	4.6 ± 0.1	17.27 ± 0.95	16.60 ± 1.00	16.86	1.23 ± 0.01	1.42 ± 0.11
	Heavy	0.55 ± 0.01	4.6 ± 0.2	17.26 ± 0.91	16.72 ± 1.01	17.11		
298	Light	0.61 ± 0.01	4.5 ± 0.1	16.78 ± 0.95	16.18 ± 1.00	16.92	1.19±0.01	1.43 ± 0.11
	Heavy	0.51 ± 0.01	4.5 ± 0.3	16.77 ± 0.91	16.28 ± 1.06	17.13		
303	Light	0.45 ± 0.01	4.1 ± 0.2	16.28 ± 0.95	15.91 ± 1.05	17.04	1.20 ± 0.02	1.25 ± 0.07
	Heavy	0.37 ± 0.01	4.0 ± 0.2	16.26 ± 0.91	16.02 ± 1.01	17.18		
308	Light	0.42 ± 0.01	3.8 ± 0.3	17.09 ± 0.81	16.80 ± 0.96	17.07	1.18 ± 0.02	1.25 ± 0.17
	Heavy	0.35±0.01	3.7 ± 0.4	17.07 ± 0.85	16.91 ± 1.05	17.21		
313	Light	0.38±0.01	3.7 ± 0.3	16.49 ± 1.01	16.28 ± 1.16	17.15	1.08 ± 0.01	1.13 ± 0.13
	Heavy	0.35 ± 0.01	3.6 ± 0.5	16.47 ± 1.05	16.33 ± 1.30	17.23		
318	Light	0.33 ± 0.01	3.5 ± 0.2	16.59 ± 1.11	16.51 ± 1.21	17.30	1.02 ± 0.02	1.01 ± 0.05
	Heavy	0.32 ± 0.01	3.5 ± 0.4	16.58 ± 1.15	16.52 ± 1.35	17.31		
323	Light	0.27 ± 0.01	3.3 ± 0.5	16.39 ± 1.01	16.47±1.26	17.45	1.01 ± 0.02	1.06 ± 0.05
	Heavy	0.26 ± 0.01	3.3 ± 0.7	16.38 ± 1.05	16.48 ± 1.40	17.48		

**Table S6.** $\frac{\partial \gamma}{\partial T}$ (in K^{-1}) values for light and heavy versions of BsADH for isopropanol and benzyl alcohol substrate.				
	isopropanol		benzyl alcohol	
	Light	Heavy	Light	Heavy
$\frac{\partial \gamma}{\partial T} \cdot 10^3$	-4.0	-4.2	-13.6	-9.1

**Table S7.** Critical distances between the substrate benzyl alcohol and some key residues at two different temperatures, 293 K and 323 K. Distances are provided in angstroms.		
	**T (K)**	
**distances**	**293**	**323**
d(C_{β}Thr40-C5$_{subs}$)	4.3 ± 0.3	4.1 ± 0.2
d(C_{β}Thr40-C6$_{subs}$)	4.4 ± 0.2	4.0 ± 0.2
d(C_{Z2}Thr49-C4$_{subs}$)	4.5 ± 0.2	4.3 ± 0.2
d(C_{H2}Thr49-C4$_{subs}$)	4.1 ± 0.2	4.4 ± 0.2
d(C_{Z2}Thr49-C5$_{subs}$)	3.8 ± 0.2	4.0 ± 0.2
d(C_{H2}Thr49-C5$_{subs}$)	3.9 ± 0.2	4.4 ± 0.3
d(C_{δ}Leu262-C5$_{subs}$)	3.7 ± 0.2	3.8 ± 0.2
d(C_{δ}Leu262-C6$_{subs}$)	4.2 ± 0.3	4.3 ± 0.3
d(C_{γ}Val286-C3$_{subs}$)	4.1 ± 0.2	4.2 ± 0.2
d(C_{β}Val286-C3$_{subs}$)	5.2 ± 0.2	5.4 ± 0.2

Table S8. Steady state turnover numbers (k_{cat}) of light and ^{13}C, ^{15}N-heavy BsADH with various substrates at 20 °C and 50 °C.

**Substrate**	**Light enzyme**	**Heavy enzyme**	**Light enzyme**	**Heavy enzyme**
	**At 20 °C** $\boldsymbol{k}_{\mathbf{cat}}$ (s^{-1})		**At 50 °C** $\boldsymbol{k}_{\mathbf{cat}}$ (s^{-1})	
"**Good**" substrates: small and non-conjugated				
Isopropanol	8.36 ± 0.73	8.18 ± 0.95	83.46 ± 3.5	80.34 ± 2.48
2-Butanol	8.59 ± 0.67	8.28 ± 0.73	47.06 ± 4.9	48.97 ± 6.05
Ethanol	4.36 ± 0.44	3.65 ± 0.16	52.37 ± 0.22	56.82 ± 0.24
1-Pentanol	2.07 ± 0.10	1.08 ± 0.07	31.23 ± 0.10	29.56 ± 0.07
"**Bad**" substrates: bulky and/or highly conjugated				
Cyclopentanol	1.52 ± 0.22	1.15 ± 0.10	34. 43 ± 0.14	33.20 ± 0.07
Cinnamyl alcohol	1.34 ± 0.24	0.99 ± 0.22	nd	nd
Benzyl alcohol	1.09 ± 0.20	0.77 ± 0.18	10.69 ± 0.35	10.08 ± 0.30

nd: not determined

Table S9. Heavy enzyme kinetic isotope effects at 20 °C and 50 °C during catalysis by light and heavy BsADH at pH 7 under steady state conditions.

**Substrate**	**EKIE at 20 °C**	**EKIE at 50 °C**
"**Good**" substrates: small and non-conjugated		
Isopropanol	1.02 ± 0.02	1.04 ± 0.05
2-Butanol	1.04 ± 0.02	0.96 ± 0.02
Ethanol	1.19 ± 0.002	0.92 ± 0.02
1-Pentanol	1.23 ± 0.03	1.06 ± 0.07
"**Bad**" substrates: bulky and/or highly conjugated		
Cyclopentanol	1.32 ± 0.03	1.04 ± 0.02
Cinnamyl alcohol	1.42 ± 0.03	nd
Benzyl alcohol	1.42 ± 0.11	1.06 ± 0.05

nd: not determined

References

[1] J.-J. Ge, C.-Z. Yao, M.-M. Wang, H.-X. Zheng, Y.-B. Kang, Y. Li, *Org. Lett.* **2016**, *18*, 228-231.

[2] C. Ceccarelli, Z. X. Liang, M. Strickler, G. Prehna, B. M. Goldstein, J. P. Klinman, B. J. Bahnson, *Biochemistry* **2004**, *43*, 5266-5277.

[3] H. Li, W. H. Hallows, J. S. Punzi, K. W. Pankiewicz, K. A. Watanabe, B. M. Goldstein, *Biochemistry* **1994**, *33*, 11734-11744.

[4] a) M. H. M. Olsson, C. R. Sondergaard, M. Rostkowski, J. H. Jensen, *J. Chem. Theory Comput.* **2011**, *7*, 525-537; b) C. R. Sondergaard, M. H. M. Olsson, M. Rostkowski, J. H. Jensen, *J. Chem. Theory Comput.* **2011**, *7*, 2284-2295.

[5] J. C. Phillips, R. Braun, W. Wang, J. Gumbart, E. Tajkhorshid, E. Villa, C. Chipot, R. D. Skeel, L. Kale, K. Schulten, *J. Comput. Chem.* **2005**, *26*, 1781-1802.

[6] U. C. Singh, P. A. Kollman, *J. Comput. Chem.* **1986**, *7*, 718-730.

[7] M. J. S. Dewar, E. G. Zoebisch, E. F. Healy, J. J. P. Stewart, *J. Am. Chem. Soc.* **1985**, *107*, 3902-3909.

[8] D. Doron, D. T. Major, A. Kohen, W. Thiel, X. Wu, *J. Chem. Theory Comput.* **2011**, *7*, 3420-3437.

[9] W. L. Jorgensen, J. Tiradorives, *J. Am. Chem. Soc.* **1988**, *110*, 1657-1666.

[10] W. L. Jorgensen, J. Chandrasekhar, J. D. Madura, R. W. Impey, M. L. Klein, *J. Chem. Phys.* **1983**, *79*, 926-935.

[11] B. Roux, *Comput Phys Commun* **1995**, *91*, 275-282.

[12] G. M. Torrie, J. P. Valleau, *J Comput Phys* **1977**, *23*, 187-199.

[13] S. Kumar, D. Bouzida, R. H. Swendsen, P. A. Kollman, J. M. Rosenberg, *J. Comput. Chem.* **1992**, *13*, 1011-1021.

[14] M. G. Paterlini, D. M. Ferguson, *Chem. Phys.* **1998**, *236*, 243-252.

[15] B. J. Gertner, K. R. Wilson, J. T. Hynes, *J. Chem. Phys.* **1989**, *90*, 3537-3558.

[16] a) R. F. Grote, J. T. Hynes, *J. Chem. Phys.* **1980**, *73*, 2715-2732; b) J. T. Hynes, *The Theory of Chemical Reaction Dynamics, Vol. IV*, Boca Raton, FL, **1985**.

[17] H. J. Kim, J. T. Hynes, *J. Am. Chem. Soc.* **1992**, *114*, 10508-10528.

[18] M. J. Field, M. Albe, C. Bret, F. Proust-De Martin, A. Thomas, *J. Comput. Chem.* **2000**, *21*, 1088-1100.

[19] M. Roca, M. Oliva, R. Castillo, V. Moliner, I. Tuñón, *Chem-Eur J* **2010**, *16*, 11399-11411.

[20] L. Y. P. Luk, J. J. Ruiz-Pernia, W. M. Dawson, M. Roca, E. J. Loveridge, D. R. Glowacki, J. N. Harvey, A. J. Mulholland, I. Tuñón, V. Moliner, R. K. Allemann, *Proc. Natl. Acad. Sci. U. S. A.* **2013**, *110*, 16344-16349.

[21] a) C. Alhambra, J. Corchado, M. L. Sanchez, M. Garcia-Viloca, J. Gao, D. G. Truhlar, *J. Phys. Chem. B* **2001**, *105*, 11326-11340; b) D. G. Truhlar, J. L. Gao, C. Alhambra, M. Garcia-Viloca, J. Corchado, M. L. Sanchez, J. Villa, *Acc. Chem. Res.* **2002**, *35*, 341-349; c) D. G. Truhlar, J. L. Gao, M. Garcia-Viloca, C. Alhambra, J. Corchado, M. L. Sanchez, T. D. Poulsen, *Int. J. Quantum Chem.* **2004**, *100*, 1136-1152.

[22] a) J. Z. Pu, J. L. Gao, D. G. Truhlar, *Chem. Rev.* **2006**, *106*, 3140-3169; b) J. Y. Pang, J. Z. Pu, J. L. Gao, D. G. Truhlar, R. K. Allemann, *J. Am. Chem. Soc.* **2006**, *128*, 8015-8023; c) M. Garcia-Viloca, D. G. Truhlar, J. L. Gao, *Biochemistry* **2003**, *42*, 13558-13575.

[23] a) J. J. Ruiz-Pernia, L. Y. P. Luk, R. Garcia-Meseguer, S. Marti, E. J. Loveridge, I. Tuñón, V. Moliner, R. K. Allemann, *J. Am. Chem. Soc.* **2013**, *135*, 18689-18696; b) L. Y. P. Luk, J. J. Ruiz-Pernia, W. M. Dawson, E. J. Loveridge, I. Tunon, V. Moliner, R. K. Allemann, *J Am Chem Soc* **2014**, *136*, 17317-17323; c) J. J. Ruiz-Pernia, E. Behiry, L. Y. P. Luk, E. J. Loveridge, I. Tuñón, V. Moliner, R. K. Allemann, *Chem. Sci.* **2016**, *7*, 3248-3255; d) L. Y. P. Luk, J. J. Ruiz-Pernía, A. S. Adesina, E. J. Loveridge, I. Tuñón, V. Moliner, R. K. Allemann, *Angew. Chem.* **2015**, *54*, 9016-9020.
